# Acetate Metabolism in Urological Health and Disease

**DOI:** 10.17161/sjm.v1i1.23080

**Published:** 2024-12-06

**Authors:** Jianhua Xiong

**Affiliations:** 1 Department of Urology, Emory University School of Medicine, Atlanta, GA, USA;; 2 Winship Cancer Institute of Emory University, Atlanta, GA, USA;

**Keywords:** acetate, bladder, kidney, prostate cancer, urological health

## Abstract

Acetate, the simplest short-chain fatty acid, serves key roles in urological health as both a nutrient and an endogenous molecule. It originates from gut microbial fermentation of dietary fiber and is found in common foods like dairy products, pasta, coffee, and vinegar. Additionally, acetate is synthesized internally to support energy metabolism and cell signaling pathways. This review highlights recent advancements in understanding acetate’s function as a signaling molecule in regulating cell fate and activity, with implications for urological health and disease treatment. The potential of acetate as a biomarker for urological health is examined, offering valuable insights that could enhance strategies for disease diagnosis, management, and therapeutic development. Furthermore, the review explores the application of non-invasive imaging techniques to monitor acetate metabolism for the diagnosis, staging, and management of urological cancers.

Acetate has been known since ancient times, largely due to the use of vinegar in food preservation and cooking. In 1845, German chemist Hermann Kolbe made a significant breakthrough by synthesizing acetic acid from inorganic compounds for the first time [1,2]. Acetate refers to the ion formed when a proton (H^+^) is lost from acetic acid, resulting in a simple two-carbon molecule (CH_3_COO^−^). The term “acetate” can also denote salt containing this anion or an ester of acetic acid [1,2]. As the simplest of the short-chain fatty acids (SCFAs), acetate is a two-carbon (C2) molecule that plays an important role in various biological processes [3–5] (Figure 1). The levels of acetate in human tissues are influenced by both exogenous sources, such as dietary intake, and endogenous production pathways. According to the Codex General Standard for Food Additives, primary dietary sources of acetate include bread, dairy products, dried pasta, coffee and its substitutes, liquid eggs, processed meats, smoked or frozen fish, salt substitutes, ethanol, and vinegar [4,5]. Beyond dietary intake, acetate is also produced within cells through several metabolic pathways: histone deacetylation reactions, direct conversion from pyruvate in cells with high glycolytic activity, and hydrolysis of acetylated metabolites [6–11]. For example, the enzyme acyl-CoA thioesterase 12 (ACOT12) activates in the liver, releasing free acetate from acetyl-CoA, which then enters circulation to supply other tissues with a readily available metabolic substrate [9–11].

In mammals, plasma acetate is primarily produced by the gut microbiota through the fermentation of dietary fiber. This fermentation generates high concentrations of SCFAs—acetate (C2), propionate (C3), and butyrate (C4)—in a consistent ratio of 3:1:1, resulting in a combined concentration of 50–150 mM [12,13]. SCFAs are efficiently absorbed from the gut lumen and initially taken up by colonic epithelial cells [14]. The remaining SCFAs enter the liver via the portal vein, where acetate levels are further enriched as hepatocytes metabolize propionate, a gluconeogenic precursor [15]. The residual acetate is released into the bloodstream, where its concentration typically ranges from 50 μM to 200 μM in human plasma. Circulating acetate is then available for uptake and oxidation by peripheral tissues, including those in the urological system [5].

This review examines acetate’s role as a signaling molecule in urological health and disease treatment (Figure 2A). Urinary acetate levels in both healthy and diseased urological conditions are also discussed (Figure 2B). Additionally, it explores the use of non-invasive positron emission tomography (PET) imaging to assess acetate uptake and metabolism by urological cells as a tool for diagnosis, cancer staging, and monitoring of disease progression, treatment response, and therapeutic efficacy (Figure 2C).

## Role of Acetate as a Signaling Molecule in Urological Health and Pathology

**Figure 1.**
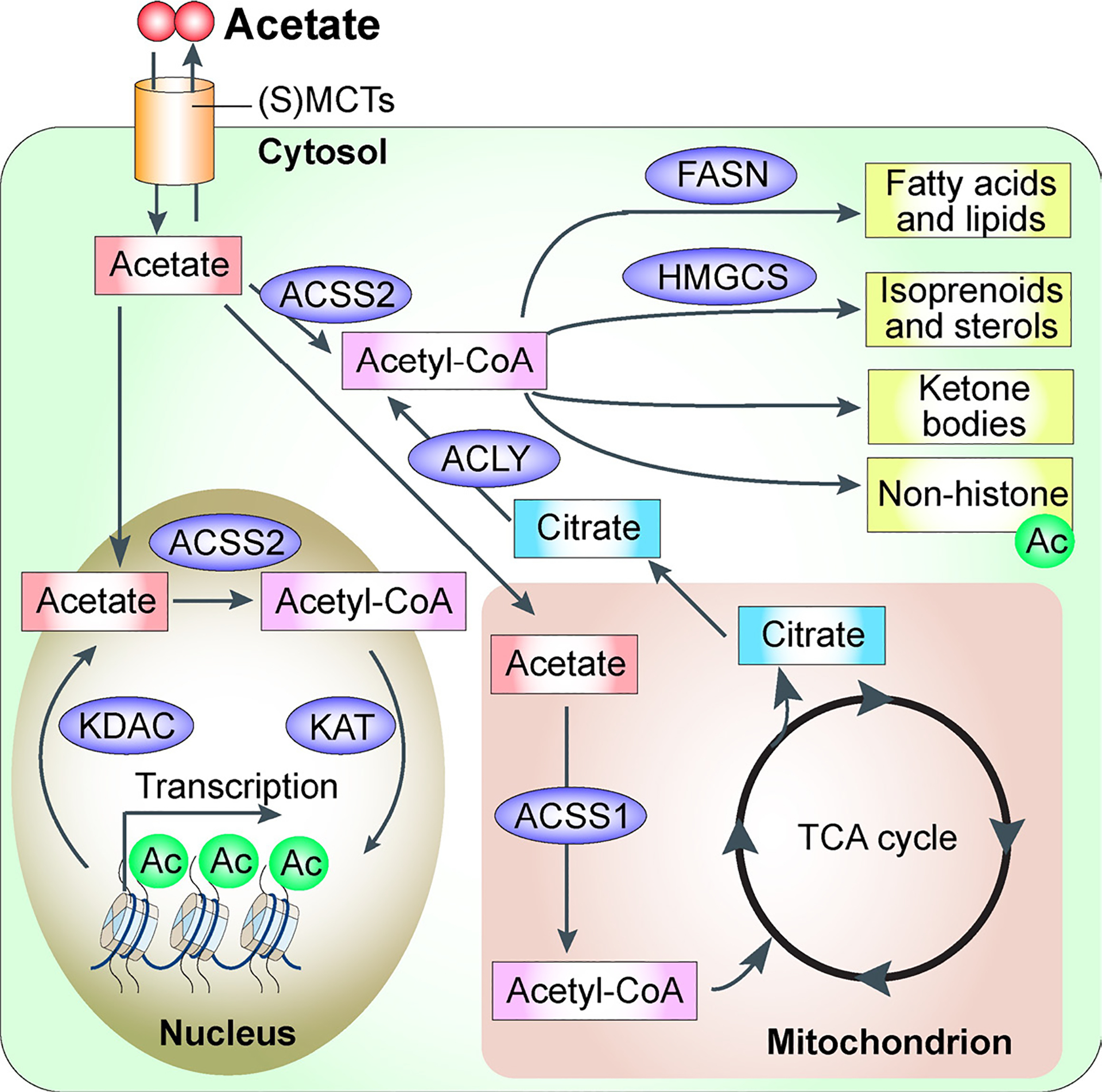
Overview of Intracellular Pathways of Acetate Metabolism in Mammalian Cells. Acetate and its derivative, acetyl-CoA, play a critical role in maintaining cellular homeostasis and regulating functions across various pathways within the cell, including those in the mitochondrion, cytosol, and nucleus. Abbreviations: Ac, acetylation; ACLY, ATP Citrate Lyase; ACSS1, Acyl-CoA Synthetase Short-Chain Family Member 1; ACSS2, Acyl-CoA Synthetase Short-Chain Family Member 2; FASN, Fatty Acid Synthase; HMGCS, 3-Hydroxy-3-Methylglutaryl-CoA Synthase; KAT, Lysine Acetyltransferase; KDAC, Lysine Deacetylase; (S)MCT, (sodium-coupled) monocarboxylate transporter.

**Figure 2.**
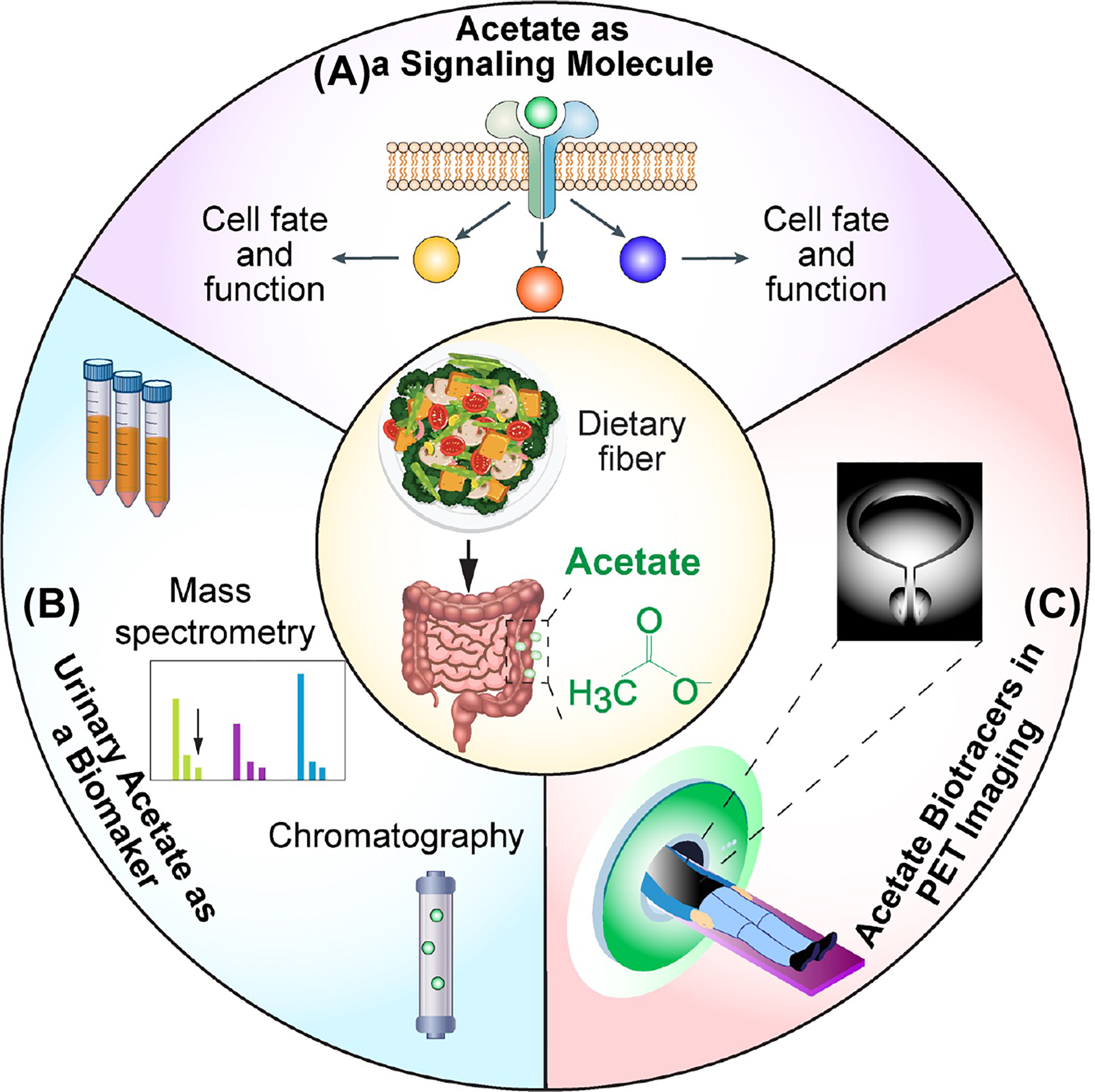
A Comprehensive Review of Acetate Metabolism in Urological Health and Disease. (A) Role of Acetate as a Signaling Molecule in Urological Health and Pathology: Acetate plays a critical role in cellular processes, including lipid biosynthesis, protein acetylation, and immune response modulation, with significant implications for cancer progression and resistance to therapy. (B) Urinary Acetate in Urological Health and Disease: Acetate holds promise as a biomarker for renal function, therapeutic monitoring, and disease management. It also demonstrates protective effects against chemotherapy-induced toxicities and kidney injuries. (C) Acetate Bio-tracers in PET Imaging for Urological Oncology and Noncancer Functional Assessment: The diagnostic utility of [^11^C]-acetate lies in imaging metabolic activity in prostate, bladder, and renal cancers, while also serving in the assessment of kidney function and ischemic nephropathies.

The physiological role of acetate extends well beyond its function as a nutritional source, with emerging evidence highlighting its importance as a signaling molecule [3,16–18] (Figure 2A). This role is mediated primarily through its conversion into acetyl-coenzyme A (acetyl-CoA) by acetyl-CoA synthetases, which catalyze the ATP-dependent ligation of acetate and CoA. Acetyl-CoA is a pivotal metabolite at the intersection of glycolysis and the TCA cycle (tricarboxylic acid cycle), playing a central role in energy metabolism [3,5,16–19] (Figure 1). Beyond this, acetyl-CoA serves as a critical substrate for numerous biosynthetic pathways, including the production of sterols, hexosamines, and ketones. Acetate also contributes significantly to fatty acid synthesis, a process involving three enzymatic steps: the formation of acetyl-CoA by acetyl-CoA synthetase 2 (ACSS2), the carboxylation of acetyl-CoA by acetyl-CoA carboxylase-α (ACCα, also known as ACC1), and the condensation of acetyl-CoA and/or malonyl-CoA by fatty acid synthase (FASN) [5] (Figure 1). Beyond its metabolic functions, acetyl-CoA acts as a central intermediate linking catabolic and anabolic pathways and serves as a second messenger influencing cell fate and function [16]. Specifically, it is the sole donor of acetyl groups for protein acetylation, a key post-translational modification that regulates protein activity and drives crucial cellular processes [17–20] (Figure 1).

A study utilizing functional genomics, comparative metabolomics, and lipidomics highlights acetate’s pivotal role in driving lipid biomass production within the complex prostate tumor microenvironment [21]. Additionally, recent findings reveal the essential involvement of acetate metabolism in neuroendocrine differentiation-mediated castration-resistant prostate cancer [22]. Together, these studies suggest that acetate metabolism plays a critical role in fostering tumor aggressiveness and metastasis in prostate cancer tissues and cell lines. In contrast, the process of epithelial-to-mesenchymal transition (EMT) - where polarized epithelial cells lose their epithelial characteristics and adopt a mesenchymal, spindle-like phenotype - is strongly linked to chemotherapy resistance and metastasis in prostate cancer [23,24]. Our recent studies, along with others, show that acetate significantly inhibits EMT and endothelial-to-mesenchymal transition (EndoMT, a specific form of EMT), while also enhancing T-cell survival and effector function [25–31]. This potentiates antitumor immunity in various cancers, suggesting that acetate’s role in prostate cancer aggressiveness may be context-dependent, with implications for therapeutic strategies.

Acetate also serves as a ligand for a family of G protein-coupled receptors known as free fatty acid receptors (FFARs) [5,32]. Among these, FFAR2 and FFAR3 are activated by SCFAs, with FFAR2 being particularly sensitive to acetate [32]. A screening of transforming genes in surgically resected gallbladder cancer indicated that FFAR2 may play an oncogenic role [33]. Interestingly, resistance to cisplatin, a key chemotherapeutic agent for metastatic bladder cancer, is often driven by elevated levels of glucose-derived endogenous acetate and its key enzyme, acetyl-CoA synthetase 2 [34]. These factors contribute to cisplatin resistance in bladder cancer cells, highlighting the potential role of acetate in cancer therapy resistance.

On the other hand, acetate has been shown to alleviate cisplatin-induced kidney injury [35]. Acetate, or vinegar (which is metabolized to acetate by gut microbiota), also reduces hyperoxaluria-induced kidney damage and improves gut microbiota and metabolomic profiles by inhibiting macrophage infiltration via the miR-493–3p/MIF axis [36,37]. Additionally, acetate mitigates sepsis-induced acute kidney injury through the inhibition of NADPH oxidase signaling in T cells [38] and reduces kidney fibrosis in an oxidative stress-dependent manner [39]. Acetate metabolism has also been shown to serve as a reno-protective agent following kidney ischemia and in hypothermically perfused kidneys [40,41]. Supplementing acetate, either directly or through dietary fiber and nutritional therapies that support SCFA-producing bacteria, may positively influence chronic renal failure management and help mitigate renal disorders associated with conditions such as polycystic ovary syndrome, nicotine-induced cardiorenal dysmetabolism, and high-fructose insulin-resistant pregnancy [42–45]. Notably, acetate has also been found to correct metabolic acidosis in premature infants, whose renal function is immature [46,47].

In addition to its renal benefits, acetate has emerged as a promising intervention for mitigating lead-induced sexual dysfunction. This effect is attributed to its ability to enhance testosterone-driven eNOS/NO/cGMP signaling and activate the Nrf2/HO-1 pathways [48]. Research indicates that sodium acetate treatment effectively lowers lead accumulation in penile tissue, as well as reduces oxidative stress markers, including malondialdehyde, oxidized glutathione levels, and acetylcholinesterase activity. Furthermore, acetate treatment restores sexual function by improving key parameters such as amount, intromission, ejaculation latencies, and frequency, effectively reversing the detrimental effects of lead exposure [48].

## Urinary Acetate in Urological Health and Disease

Acetate plays a vital role not only in addressing sexual dysfunction but also in managing medical toxicities, particularly through urine alkalinization [49,50]. A prime example is methotrexate, an antifolate commonly used in treating urological cancers, which often induces toxicity. This toxicity can be mitigated by urine alkalinization, a process in which sodium acetate is particularly effective. By alkalinizing urine in patients receiving high doses of methotrexate, sodium acetate helps reduce these adverse effects [46]. Additionally, acetate has been shown to inhibit urothelial cell proliferation, offering potential therapeutic benefits for augmentation cystoplasty [51–53].

Urinary acetate levels can be measured using advanced techniques such as gas chromatography-mass spectrometry or ion chromatography [54,55]. Noninvasive monitoring of acetate concentrations in urine serves as a valuable biomarker for various clinical applications (Figure 2B). These include assessing exposure to ischemic reperfusion injury, predicting renal changes following cold ischemia and transplantation, and tracking alcohol oxidation [56–58]. These findings highlight the broader clinical utility of acetate in urological health, paving the way for further exploration of diagnostic and therapeutic applications.

## Acetate Bio-tracers in PET Imaging for Urological Oncology and Noncancer Functional Assessment

Building on acetate’s role in urinary toxicities and as a biomarker, its use as a bio-tracer in PET imaging offers valuable insights into both urological oncology and noncancer functional assessments. [^18^F]-Fluorodeoxyglucose ([^18^F]-FDG) remains the most widely used bio-tracer in clinical PET imaging, primarily due to its effectiveness in providing metabolic activity readouts. It has proven highly effective in identifying rapidly proliferating tumors with high glucose uptake [59, 60]. However, [^18^F]-FDG has limitations: many tumors do not exhibit elevated glucose consumption, and some face restricted glucose availability due to abnormal vasculature [5,61]. As an alternative, [^11^C]-acetate shows significant promise in addressing these gaps [62]. This tracer has proven especially effective in prostate cancer imaging, assisting with tumor localization, detecting early recurrence, guiding adaptive radiotherapy, and visualizing bone metastases in advanced cases [63–74]. Additionally, [^11^C]-acetate has demonstrated value in imaging bladder and renal cancers [75–81]. Its applications across urological oncology underscore its utility for non-invasive, dynamic assessments of functional and metabolic parameters.

In healthy individuals, [^11^C]-acetate PET imaging enables direct measurement of renal oxygen consumption and tissue perfusion across a range of perfusion levels, providing valuable insights into kidney function. While this technique has yet to be validated in patients with chronic kidney disease, it holds promise for evaluating ischemic nephropathies in individuals considered for revascularization [82]. Beyond clinical applications, [^11^C]-acetate is also a powerful tool for in vivo metabolic research. Recent studies in mouse prostate cancer models demonstrate that late-phase [^11^C]-acetate PET-imaging offers enhanced contrast, with tracer accumulation in lipid fractions that reveal insights into acetate-dependent lipogenesis [83]. This versatility underscores [^11^C]-acetate’s potential to enhance our understanding of urological cancers, metabolic diseases in urology, and related conditions (Figure 2C).

## Prospective Directions for Future Research

This review outlines acetate’s complex roles in urological health, disease treatment, and diagnostics. However, numerous gaps remain to be addressed to fully harness acetate’s therapeutic and diagnostic potential. Below are several prospective research directions that could significantly advance this field:

### Molecular Mechanisms of Acetate in the Tumor Microenvironment and Therapy Resistance:

Future studies should explore the molecular mechanisms through which acetate influences tumor metabolism, particularly in prostate and bladder cancers. Understanding how acetate impacts pathways such as EMT, immune modulation, and chemoresistance could shed light on its dual role in both tumor progression and therapy resistance. Investigating the context-dependent effects of acetate on cancer aggressiveness and metastasis may enable the development of targeted strategies that inhibit pro-cancerous actions while enhancing immune function and reducing drug resistance associated with EMT. Recent research has emphasized the critical role of acetate in tumor cell interactions, particularly in nutrient-limited environments, where tumor cells capture acetate as a carbon source to meet both catabolic and anabolic demands [19,21,84–86]. This highlights the potential metabolic vulnerabilities within tumors, suggesting that the intracellular mechanisms involved in generating and shuttling acetate between cells could serve as promising targets for pharmacologic therapies.

### Acetate as a Biomarker in Urological Diseases:

Additional studies are needed to validate urinary acetate as a non-invasive biomarker for early detection and progression of urological conditions, such as bladder cancer and kidney disease. Research could focus on establishing threshold values for urinary acetate that correlate with disease severity and response to treatment. Longitudinal studies assessing acetate levels in various patient populations would help refine its clinical utility as a biomarker and guide therapeutic interventions.

### Potential Targets of Acetate Metabolism in Urological Health and Disease:

Acetate metabolism presents therapeutic opportunities in urological diseases. In prostate cancer, inhibiting ACSS2 may disrupt tumor aggressiveness and lipid production, while modulating acetate levels could counteract EMT and EndoMT to reduce chemotherapy resistance and enhance antitumor immunity. In bladder cancer, targeting acetate-driven resistance pathways, including those involving ACSS2, could improve cisplatin efficacy. Acetate’s reno-protective effects, such as reducing kidney injury and oxidative stress, further underscore its potential to minimize treatment side effects. Additionally, acetate’s role as an FFAR2 ligand offers pathways to modulate oncogenesis. Dietary or microbiota-based approaches to boost acetate levels may also benefit chronic kidney diseases and related metabolic disorders.

### Expanding Applications of Acetate PET Imaging:

[^11^C]-acetate PET imaging shows promise for tracking metabolic changes in prostate, bladder, and kidney cancers, yet more research is needed to expand its clinical applications. Studies could focus on the utility of [^11^C]-acetate PET imaging in monitoring therapeutic responses and progression in urological cancers, potentially providing a non-invasive alternative to traditional biopsies. Additionally, evaluating the sensitivity and specificity of [^11^C]-acetate PET imaging in non-cancer conditions, such as ischemic nephropathies and renal perfusion disorders, could broaden its clinical relevance.

### Interplay Between Gut Microbiota and Urological Health:

The role of microbiota-derived acetate in urological health and disease remains underexplored. Future studies could investigate how dietary interventions, probiotics, and prebiotics that enhance acetate production impact urological health. Research in this area could establish a link between gut-derived acetate and its protective or detrimental effects on renal, prostate, and bladder tissues, providing new insights into diet-based preventive strategies and therapies for urological diseases.

### Exploring Acetate’s Role in Sexual Dysfunction and Renal Toxicities:

Further studies should delve into acetate’s mechanisms in mitigating toxicities, such as methotrexate-induced kidney injury and lead-induced sexual dysfunction. Understanding how acetate contributes to these protective effects on a cellular level could open avenues for therapeutic applications across broader urological conditions, particularly in addressing renal toxicities associated with common urological treatments.

### Preclinical and Clinical Trials for Acetate Supplementation:

Clinical trials assessing the impact of acetate supplementation in various urological conditions are warranted. This includes evaluating the benefits of dietary acetate or SCFA-producing bacteria in patients with chronic renal failure, prostate cancer, or chemotherapy. The findings from these trials could establish acetate supplementation as a feasible, adjunctive strategy for managing urological diseases and enhancing patient outcomes.

Advancing knowledge in these areas will help unravel the complexities of acetate metabolism and its applications in urological health and disease, with the goal of translating findings into actionable therapies and diagnostics.
